# Supplementary material for: Analysis of medico-legal claims related to deliveries: Caesarean section vs. vaginal delivery
Source: PLoS One. 2024 Nov 19;19(11):e0312614. doi: 10.1371/journal.pone.0312614 (PMC11575767; doi:10.1371/journal.pone.0312614)
Supplement: Attachment — Submitted filename: Reviewers comments to the Plos one manuscript.docx [file pone.0312614.s002.docx]

**Reviewer’s comments**: Dr Herbert Kayiga

**General comments:**

I take this privilege to appreciate authors for taking off time to undertake this study. It’s in an area of great interest in clinical practice. Please address the following aspects to make it more relevant globally rather than Iran or Europe.

**Specific comments**:

**Abstract:**

**Introduction**: It would be ideal to mention the authority that said rather than “it was said….

**Methods:** I think the authors intended to mention “we analysed medico-legal cases that occurred during a two-year period. Kindly include the years to avoid guessing when the actual study occurred.

It would be prudent to indicate where exactly the study was conducted and the setting “was it in private or public health facility setting?” Who was responsible for making the claims? Are there charges implicated with the medico-legal claims? What kind of penalties are available for the implicated healthcare providers? Where there any instrumental deliveries as indicated in the settings? If yes, what proportion of women had instrumental delivery?

**Results:** I think it would have been so handy knowing how high the claims that accrued when compared to the vaginal delivery. This is however, implied. What was the reference group? What kind of neonatal complications led to the observed high claims as seen in the vaginal route? What other cadres made up for the other staffs? Are these cadres permitted to perform the caesarean sections as the obstetricians? Elaborate more on the difference between closed and paid claims to avoid confusion.

The authors mention “*However, the number of instrumental deliveries and the reasons for claims, exhibited significant differences between the two groups (P=0.021)”.* It’s not clear though the groups that are being compared. How many instrumental deliveries occurred in the caesarean vs. vaginal groups if I can assume that these are the comparative groups?

**The conclusion**: “The study concluded that caesarean section did not result in an increased risk of medicolegal claims or payment compensation compared to vaginal delivery”, doesn’t appear to be arising from the work. If caesarean section constituted high maternal complications than vaginal delivery, why is it then that it didn’t have higher claims? What complication rates were sufficient to constitute a claim?

**Introduction/Background**:

Is it the global perspective that all obstetricians/gynaecologists face higher medico-legal claims more other physicians or a context-based observation? In some countries, surgeons are more at risk than the obstetricians.

I think to would be prudent to mention the context or setting of where the reduced mortality following c/sections is observed because in low resource settings, the cost and mortality is way higher following caesarean delivery than observed for vaginal delivery.

What is the global caesarean section rate? I would be nice if wherever there’s a comparison, the reference range is mentioned.

What is the setting like in Iran? Are there any other cadres allowed to do caesarean sections other than obstetricians? Who conducts vaginal deliveries? Is it still the obstetricians or even the midwives do the job? I would be ideal helping the readership to have glimpse of the delivery processes in Iran. Are the claims similar for elective vs. emergent caesareans? It would have been nice to know of the implications of these medico-legal claims in Iran. Try to introduce the concept of paid and closed claims as indicated in the abstract highlighting the difference.

What authority determines the nature of claim that arises? Is it the legal fraternity or the medical council? It is so informative if you can help the readership view the impact through the lenses of the Iranian medico-legal environment.

**Methods**: I think it’s best to call it methods rather than method. Please add these years in the abstract write up. It would be nice to separate the study design and the ethical approvals. Write these subsections differently and add more details in each of them.

Who were the participants? Was it the patients or the affected family members? Was it a document review process or you conducted participant interviews? How did you minimize recall biases since the interviews or document review was 3-5 years after the incidences? How did you identify the potential participants? What measures were put in place to ensure that patient safety was observed? At what point were the discussions held? Was it after the hospital admissions or after discharge from the health facilities? What is the setting of the deliveries? Who takes the mandate to determine whether instrumental, vaginal or caesarean section is to be taken? How do the patients get to obtain care in the Iranian setting? Is at the jurisdiction of the Ministry of Health or the patients? Who determines whether the care accorded to the patient is optimal or not so as to determine to proceed to the next phase of making a legal claim?

What was the baseline from which these documents were drawn? I see the other cadres constituted anaesthetists, and midwives. These all have different responsibilities in hospital setting. Did you expect them to have the same weight in terms of claims? It also appears like the cases were obstetric in nature, what kind of gynaecological causes attracted attention in the study period? Were there any other physicians like GPs, Surgeons who had cases in this study period because some of the patients had bowel or bladder injuries?

Kindly add a write up on the study settings. It’s not clear who made the decisions on the wards, who runs the care of the patients. Are the claims more in private than the public settings? Is there any correlation between the patient volumes and the legal claims? Who determine the nature of the claims? How does a medical case become a medico-legal claim? Who files the claims? Is it the patients, their lawyers or the hospital that files a claim? How do the cases get up in court? What kind of penalties are issues in court?

The inclusion and exclusion criteria are not mentioned in the write up. Clarity is needed to determine the proportion of deliveries that turned out to be claims in the study period.

**Results**: I think it would nice to write more about the settings of the health facilities in the methods rather than introducing them in the result section for the first time. It’s still not clear if it’s a document review or participant interviews. Please add a table to show the baseline characteristics of the participants. It’s not so clear who the other medical staffs that were involved in the study. What participant characteristic were more likely to make a claim? Prime gravida or multiparous? Best in the introduction of the results section to mention how many deliveries occurred generally, how many SVDs, instrumental deliveries (vacuum or forceps) occurred in the study periods? Who assisted in the deliveries and the key aspects that contributed to a claim and a non-claim? How many closed or paid claims were reported? Were there any c/sections that were done with no indication? I don’t see any IUFD in the table? Did you regard perinatal mortality as IUFDs? Otherwise, how did you capture early neonatal deaths? The two groups being compared that were mentioned to be similar are quite confusing. Did you mean among those that malpractice vs. cases with no malpractice or vaginal vs. c/section?

What characteristics were reported among those whose claims were paid? Are these characteristics similar among those who had vaginal vs. caesarean delivery? Adding a multivariate analysis would have improved the overall impact and eliminate any confounding factors.

**Discussion**: It would be ideal to recap the overall aim of the study and mention the key significant findings before comparison with the other studies. Knowing the contribution of the other specialties in regards to the claims contributed by obs/gyn would have been so handy at this point. In the write up there are no gyn cases! So how would you conclude that obs has more legal claims than gyn even in the Iranian setting? Which region are you referring to with the CS rate of 48%? Please don’t assume that the readers see through your eyes.

Mentioning “*Additionally, the rate of paid claims among emergency caesarean sections was similar to that of vaginal deliveries*” is not the best comparison. I think I would compare it to the elective cases as the participant characteristics would be different”. I find most of the comparisons in Europe! It would have been interesting to hear how neighbouring countries to Iran or Sub-Saharan Africa would compare in regards to medico-legal claims and obstetric cases. The strength of the study is not water-tight. Having it conducted in Iran doesn’t make it a strength in my view. Is it that there are many claims following c/s in Iran or because WHO thinks the C/s rate is higher than in other settings?

The limitations are not so clear as well. How would conducting a case-control study improve the study findings for your study? What did you mean by conduction the whole province evaluation would be a limitation? What challenges did you meet in executing this study?

**Conclusion:** In my evaluation, the claim that “*this study revealed that claims related to deliveries were often associated with perinatal death*” can not be drawn from the study findings. Though instrumental delivery is claimed to be a cause of malpractice, the authors make no mention of how many instrumental deliveries were reported among the claims. Clarity needs to be made in this regard. Though the authors claim to have conducted the study to educate the specialists on medico-legal claims, there is no mention that there was a gap in the knowledge among the specialists. I request for recommendations that can be drawn from the study findings that can inform policy and future research.
