# Supplementary material for: Analysis of medico-legal claims related to deliveries: Caesarean section vs. vaginal delivery
Source: PLoS One. 2024 Nov 19;19(11):e0312614. doi: 10.1371/journal.pone.0312614 (PMC11575767; doi:10.1371/journal.pone.0312614)
Supplement: Attachment — Submitted filename: commen cesarean.docx [file pone.0312614.s003.docx]

Journal Requirements:

When submitting your revision, we need you to address these additional requirements.

1. Please ensure that your manuscript meets PLOS ONE's style requirements, including those for file naming. The PLOS ONE style templates can be found at
   https://journals.plos.org/plosone/s/file?id=wjVg/PLOSOne_formatting_sample_main_body.pdf and 
   <https://journals.plos.org/plosone/s/file?id=ba62/PLOSOne_formatting_sample_title_authors_affiliations.pdf>

Thank you. I ensured that manuscript meets PLOS ONE's style requirements

2. Thank you for submitting the above manuscript to PLOS ONE. During our internal evaluation of the manuscript, we found significant text overlap between your submission and previous work in the [introduction, conclusion, etc.].
    
   We would like to make you aware that copying extracts from previous publications, especially outside the methods section, word-for-word is unacceptable. In addition, the reproduction of text from published reports has implications for the copyright that may apply to the publications.
    
   Please revise the manuscript to rephrase the duplicated text, cite your sources, and provide details as to how the current manuscript advances on previous work. Please note that further consideration is dependent on the submission of a manuscript that addresses these concerns about the overlap in text with published work.
    
   [If the overlap is with the authors’ own works: Moreover, upon submission, authors must confirm that the manuscript, or any related manuscript, is not currently under consideration or accepted elsewhere. If related work has been submitted to PLOS ONE or elsewhere, authors must include a copy with the submitted article. Reviewers will be asked to comment on the overlap between related submissions (http://journals.plos.org/plosone/s/submission-guidelines#loc-related-manuscripts).]
    
   We will carefully review your manuscript upon resubmission and further consideration of the manuscript is dependent on the text overlap being addressed in full. Please ensure that your revision is thorough as failure to address the concerns to our satisfaction may result in your submission not being considered further.

Thank you for your feedback. I have thoroughly reviewed the entire manuscript using the plagiarism detection tool, iThenticate. I have also attached the file for your reference. Based on the results, I believe that the similarity rate is within an acceptable range, considering that there may be some common words or phrases present in the manuscript. If you have any specific concerns or questions about the detected similarities, please let me know, and I'll be happy to assist you further.

3. In this instance it seems there may be acceptable restrictions in place that prevent the public sharing of your minimal data. However, in line with our goal of ensuring long-term data availability to all interested researchers, PLOS’ Data Policy states that authors cannot be the sole named individuals responsible for ensuring data access (http://journals.plos.org/plosone/s/data-availability#loc-acceptable-data-sharing-methods).

Data requests to a non-author institutional point of contact, such as a data access or ethics committee, helps guarantee long term stability and availability of data. Providing interested researchers with a durable point of contact ensures data will be accessible even if an author changes email addresses, institutions, or becomes unavailable to answer requests.

Before we proceed with your manuscript, please also provide non-author contact information (phone/email/hyperlink) for a data access committee, ethics committee, or other institutional body to which data requests may be sent. If no institutional body is available to respond to requests for your minimal data, please consider if there any institutional representatives who did not collaborate in the study, and are not listed as authors on the manuscript, who would be able to hold the data and respond to external requests for data access? If so, please provide their contact information (i.e., email address). Please also provide details on how you will ensure persistent or long-term data storage and availability.

Thank you. I provide the information for a non-author contact person who can be reached for data access.

Name: Zahra Omidi

Email: [z.omidii67@gmail.com](mailto:z.omidii67@gmail.com)

Tel: +98 9123491650

4. PLOS requires an ORCID iD for the corresponding author in Editorial Manager on papers submitted after December 6th, 2016. Please ensure that you have an ORCID iD and that it is validated in Editorial Manager. To do this, go to ‘Update my Information’ (in the upper left-hand corner of the main menu), and click on the Fetch/Validate link next to the ORCID field. This will take you to the ORCID site and allow you to create a new iD or authenticate a pre-existing iD in Editorial Manager. Please see the following video for instructions on linking an ORCID iD to your Editorial Manager account: <https://www.youtube.com/watch?v=_xcclfuvtxQ>

Thank you. I provided the orchid id for the corresponding authors.

Is the manuscript technically sound, and do the data support the conclusions?

The manuscript must describe a technically sound piece of scientific research with data that supports the conclusions. Experiments must have been conducted rigorously, with appropriate controls, replication, and sample sizes. The conclusions must be drawn appropriately based on the data presented.

Reviewer #1: Partly

Reviewer #2: Yes

2. Has the statistical analysis been performed appropriately and rigorously?

Reviewer #1: No

Reviewer #2: Yes

3. Have the authors made all data underlying the findings in their manuscript fully available?

The [PLOS Data policy](http://www.plosone.org/static/policies.action#sharing) requires authors to make all data underlying the findings described in their manuscript fully available without restriction, with rare exception (please refer to the Data Availability Statement in the manuscript PDF file). The data should be provided as part of the manuscript or its supporting information, or deposited to a public repository. For example, in addition to summary statistics, the data points behind means, medians and variance measures should be available. If there are restrictions on publicly sharing data—e.g. participant privacy or use of data from a third party—those must be specified.

Reviewer #1: Yes

Reviewer #2: Yes

4. Is the manuscript presented in an intelligible fashion and written in standard English?

PLOS ONE does not copyedit accepted manuscripts, so the language in submitted articles must be clear, correct, and unambiguous. Any typographical or grammatical errors should be corrected at revision, so please note any specific errors here.

Reviewer #1: Yes

Reviewer #2: No

5. Review Comments to the Author

Please use the space provided to explain your answers to the questions above. You may also include additional comments for the author, including concerns about dual publication, research ethics, or publication ethics. (Please upload your review as an attachment if it exceeds 20,000 characters)

Reviewer #1: Though the authors cover a pertinent topic on medico-legal claims, the write-up needs to be improved to ensure that the grammar is right and appropriate. The manuscript needs to make a number of corrections to make it more informative to the readership.

Thank you for your comment. I have revised the manuscript to address the grammar issues.

The statistical analysis needs to be improved. A table of baseline characteristics needs to be added and preferably adding a multivariate analysis would improve the strength of the associations made by the authors.

Thank you for your comment. I have made the following revisions to the manuscript:

1. Added the baseline characteristics in Table 1.
2. Conducted a multivariable analysis to enhance the strength of the study.
3. Included the results of the regression analysis in the Results section.

The discussion is more around Europe than even the local context or SSA.

Thank you for your comment. I have revised the discussion part of the manuscript to include the results of relevant Iranian research.

The conclusions don't seem to fully arise from the study and the recommendations drawn are not from the study findings.

Thank you for your comment. I have revised the conclusion part. Regarding the recommendation, given the high number of lawsuits against OB/GYN surgeons and the importance of identifying the factors associated with these claims, our study's findings suggest that evaluating medicolegal documents could assist surgeons in being better informed and potentially reducing the risk of receiving complaints.

Reviewer #2: Dear authors, thank you for conducting an interesting topic; the findings have important implications for clinicians and public health. However, I have some comments that should be addressed before the publication
- the entire manuscripts need to be revised for grammatical errors and punctuation issues and references for some sentences are missing ( e.g. Discussion part paragraph 2 the end sentences should be added.

Thank you for your comment. I appreciate your review of this article and your interest in the topic. I have added the end sentence to the end of the second paragraph and made revisions to the manuscript accordingly.

- The authors should include statistical analysis methods in the abstract part

Thank you for your comment. I have included the statistical analysis in the abstract part of the manuscript.

- the authors in the main manuscript, stated the conducted using medico-legal dispute documents from March 2018 to February 2020. This information is better if also available in the methods section of the abstract

Thank you for your comment. I have included this information in the abstract part of the manuscript.

- also, there are some inconsistencies in your abstract and main manuscript. for example, in the abstract, we analyzed medico-legal cases that during two years, but in your result we stated .. a total of 227 women with obstetric claims, meeting the inclusion criteria, were included in this study over three years. why does difference happen? study period should be consistent

Thank you for your comment. I apologize for the mistake. I have corrected it.

- this study does not incorporate a qualitative study of the reason for claims associated with delivery from a Health care provider perspective and mothers, and other stakeholders. so, better if should be stated as the limitation of this study.

Thank you for your comment. I have incorporated this limitation into our study.

- finally. based on your findings why not suggest that public intervention should be targeted at reducing the prevalence of delivery-related claims?

Thank you for your comment. I have incorporated this conclusion into our study.
