# Supplementary material for: Analysis of medico-legal claims related to deliveries: Caesarean section vs. vaginal delivery
Source: PLoS One. 2024 Nov 19;19(11):e0312614. doi: 10.1371/journal.pone.0312614 (PMC11575767; doi:10.1371/journal.pone.0312614)
Supplement: Attachment — Submitted filename: Reviewers comments to the Plos one manuscript_version 2 (1).docx [file pone.0312614.s005.docx]

**Reviewer’s comments**: Dr Herbert Kayiga

**General comments:**

I take this privilege to appreciate authors for revising the manuscript. Most of the comments were not addressed in the current submission. I wonder whether they submitted the correct version of manuscript. Otherwise I don’t see any improvement made in the revised version.

Thank you so much for reviewing this article and for your comments. I want to sincerely apologize that I missed addressing your feedback in the previous version. To be honest, I was unable to see your comments in the prior decision letter, which led to this oversight on my part. I feel terrible about that, and I have made every effort to thoroughly incorporate your feedback in this latest revision.

Please let me know if there are any other changes or clarifications you would like me to make. I am looking forward to hearing from you. I greatly appreciate the time and consideration you have provided throughout this process.

**Specific comments**:

**Abstract:**

**Introduction**: It would be ideal to mention the authority like the WHO, FIGO or Iranian Ministry of Health said …. rather than “it was said…. I still notice the authors didn’t revise this aspect. The write up on “*Chi-square or t-tests were employed to compare factors between paid claims and closed claims”* should be put in the methods section.

Thank you for your comment. I revised it.

**Methods:** The revision has been made concerning the study period but other aspects as indicated in the reviewer’s report are not made.

See the comments before

*It would be prudent to indicate where exactly the study was conducted and the setting “was it in private or public health facility setting?” Who was responsible for making the claims? Are there charges implicated with the medico-legal claims? What kind of penalties are available for the implicated healthcare providers?*

Thank you for your comment. I have revised the text as follows: All documents were finally presented to the Iranian Medical Legal Organization. Regarding the final decision and punishment, this was named in the abstract and explained in more detail in the main manuscript.

*Where there any instrumental deliveries as indicated in the settings? If yes, what proportion of women had instrumental delivery?*

Thank you for your comment. I added this point to result.

**Results:**

It’s still quite confusing as the comments raised in the first report are not addressed. Kindly see the points below. It’s not clear whether the reference cadre were surgeons or general doctors. What claims were reported against other staffs? Clarity is needed for closed vs paid claims.

**Previous comments**;

*I think it would have been so handy knowing how high the claims that accrued when compared to the vaginal delivery. This is however, implied. What was the reference group?*

Thank you for your comment. I revised it.

*What kind of neonatal complications led to the observed high claims as seen in the vaginal route?*

Thank you for your comment. Due to statistical limitations, it was not possible to compare each type of claim between the two groups. Instead, the total number of neonatal and maternal claims was compared between the two groups.

*What other cadres made up for the other staffs? Are these cadres permitted to perform the caesarean sections as the obstetricians? Elaborate more on the difference between closed and paid claims to avoid confusion.*

Thank you for your comment. I have added information regarding other staff. Yes, in Iran, Ob/Gyn physicians do perform caesarean sections.

Thank you for your comment. I added the explanation of paid claims and closed claims to method.

*.*

*The authors mention “However, the number of instrumental deliveries and the reasons for claims, exhibited significant differences between the two groups (P=0.021)”. It’s not clear though the groups that are being compared. How many instrumental deliveries occurred in the caesarean vs. vaginal groups if I can assume that these are the comparative groups?*

Thank you for your comment. In this study, all instrumental deliveries were related to vaginal deliveries, and I have added the number of these deliveries

**The conclusion**: I notice that comments were still not addressed.

“The study concluded that caesarean section did not result in an increased risk of medicolegal claims or payment compensation compared to vaginal delivery”, doesn’t appear to be arising from the work. If caesarean section constituted high maternal complications than vaginal delivery, why is it then that it didn’t have higher claims? What complication rates were sufficient to constitute a claim?

Thank you for your comment. While the total number of all deliveries in this study was not provided, the proportion of paid claims aligned with the recent percent of vaginal deliveries and Cesarean sections in Iran. However, further study is still needed. I have revised the conclusion accordingly.

**Introduction/Background**:

I notice the comments raised are not addressed as expected. I wonder whether authors really saw the comments in this area.

Thank you for your detailed comments. I truly appreciate the feedback you provided. I apologize that I missed addressing them in the previous version - I am grateful for the opportunity to correct my mistakes.

The global C/s rate is not mentioned. The role of other staffs is not clear and how they get implicated is not clear. More clarity is needed on the contextual setting in the private and public facilities in Iran in regards to the delivery processes.

See comments below;

*Is it the global perspective that all obstetricians/gynaecologists face higher medico-legal claims more other physicians or a context-based observation? In some countries, surgeons are more at risk than the obstetricians.*

Thank you for your comment. I have revised the text and provided information about three countries that reported higher rates of lawsuits in the obstetrics and gynecology specialty compared to most other medical specialties.

*I think to would be prudent to mention the context or setting of where the reduced mortality following c/sections is observed because in low resource settings, the cost and mortality is way higher following caesarean delivery than observed for vaginal delivery.*

Thank you for your comment. In the introduction, I explained the results of a previous study that evaluated the relationship between cesarean section and maternal mortality globally over the past decade.

*What is the global caesarean section rate? I would be nice if wherever there’s a comparison, the reference range is mentioned.*

Thank you for your feedback. I have updated the sentence based on your suggestion

*What is the setting like in Iran? Are there any other cadres allowed to do caesarean sections other than obstetricians? Who conducts vaginal deliveries? Is it still the obstetricians or even the midwives do the job? I would be ideal helping the readership to have glimpse of the delivery processes in Iran. Are the claims similar for elective vs. emergent caesareans? It would have been nice to know of the implications of these medico-legal claims in Iran. Try to introduce the concept of paid and closed claims as indicated in the abstract highlighting the difference.*

Thank you for your comment.

In Iran, cesarean sections can be performed by obstetrician-gynecologists.

For vaginal deliveries, these are typically conducted by obstetricians, but midwives also play a significant role, especially for low-risk births.

For both emergency and planned caesarean sections, patients could potentially make legal claims against their surgeon, and in such cases all relevant medical-legal documents would be carefully evaluated.

Paid claims represent successful malpractice lawsuits where the healthcare provider or insurer made a financial payout to the plaintiff, while closed claims encompass the denied/dismissed claims.

I added these points to the method section.

*What authority determines the nature of claim that arises? Is it the legal fraternity or the medical council? It is so informative if you can help the readership view the impact through the lenses of the Iranian medico-legal environment.*

Thank you for your comment. In the Iranian medico-legal framework, finally, all relevant medical-legal documents pertaining to malpractice claims are presented to the Iranian Medical Legal Organization, the main regulatory body overseeing the medical profession. The medical experts carefully evaluate these documents to assess the merits of the claim.

I added these points to the method section.

**Methods**: Comments not addressed. I pray the authors submitted the right version for the review! No effort was made to address the concerns raised.

Thank you for your feedback. I apologize for missing these comments in my previous revision. I'm grateful for the opportunity to revise the manuscript based on your feedback.

**Comments submitted before**:

*I think it’s best to call it methods rather than method. Please add these years in the abstract write up. It would be nice to separate the study design and the ethical approvals. Write these subsections differently and add more details in each of them.*

Thank you for the feedback. I have updated the abstract to include the years the study was conducted, and I have separated the details on ethical approval from the study design section. Additionally, I have added more specifics to different parts.

*Who were the participants? Was it the patients or the affected family members? Was it a document review process or you conducted participant interviews? How did you minimize recall biases since the interviews or document review was 3-5 years after the incidences?*

Both patients and their families could have claimed against the physicians if the patients died or suffered significant morbidity. However, in this study, we only evaluated the medicolegal documents and did not conduct any interviews with the patients' families. Normally, medicolegal documents report all the details and are fully informative.

*How did you identify the potential participants?*

Thank you for your comment. I added the details regarding inclusion criteria for including participants. The related key words to delivery clams were searched through the medicolegal documents and all cases that met the inclusion criteria were included.

*What measures were put in place to ensure that patient safety was observed? At what point were the discussions held? Was it after the hospital admissions or after discharge from the health facilities? What is the setting of the deliveries?*

Thank you for your comment. In all hospitals, whether public or private, information regarding patients is recorded in the hospital system. In the case of claims, these documents are typically presented to the medicolegal court. Usually, the cases claimed against the surgeon are filed after the patient's discharge from the hospital.

*Who takes the mandate to determine whether instrumental, vaginal or caesarean section is to be taken? How do the patients get to obtain care in the Iranian setting? Is at the jurisdiction of the Ministry of Health or the patients? Who determines whether the care accorded to the patient is optimal or not so as to determine to proceed to the next phase of making a legal claim?*

The decision regarding the mode of delivery was made by the obstetricians and gynecologists. In some cases, midwives also monitored the patients during labor. After claims were received at the Iran Legal Medical Organization, all claims were evaluated by experienced professionals. All relevant documents were transferred to them. They may have performed multiple sessions to declare their final decision.

*What was the baseline from which these documents were drawn? I see the other cadres constituted anaesthetists, and midwives. These all have different responsibilities in hospital setting. Did you expect them to have the same weight in terms of claims?*

Thank you for your comment. I divided the claims to determine the proportion of OB/GYN claims related to delivery. I also added a table in the results to show the proportion of each specialty, including midwives, for the claims related to delivery.

*It also appears like the cases were obstetric in nature, what kind of gynaecological causes attracted attention in the study period?*

Thank you for your comment. In this study, we aimed to evaluate cases that were related to delivery and obstetrics problems during this time period, not all claims against obstetricians/gynecologists.

*Were there any other physicians like GPs, Surgeons who had cases in this study period because some of the patients had bowel or bladder injuries?*

*Thank you for your comment.* I also added a table in the results to show the proportion of each specialty, including midwives, for the claims related to delivery.

*Kindly add a write up on the study settings. It’s not clear who made the decisions on the wards, who runs the care of the patients. Are the claims more in private than the public settings? Is there any correlation between the patient volumes and the legal claims?*

Thank you for your comment. In Iran like the other countries, in private and governmental hospitals, the management of patients was carried out by obstetricians and gynecologists. However, in teaching hospitals, obstetrics and gynecology residents under the supervision of obstetrician/gynecologist assistant professors managed the patients. The results of this study indicated there was no difference between the two claims regarding the different types of hospitals. It is possible that there is no correlation between patient volume and the number of legal claims. However, further studies would be required to confirm this result.

*Who determine the nature of the claims? How does a medical case become a medico-legal claim? Who files the claims? Is it the patients, their lawyers or the hospital that files a claim? How do the cases get up in court? What kind of penalties are issues in court?*

Thank you for your comment. All patients, or their family members if the patient is unable, can file claims. All relevant medical-legal documents pertaining to these malpractice claims are finally presented to the Iranian Medical Legal Organization. The commission at this organization, which includes 3 experienced professionals, conducts a detailed evaluation of each claim. The medical experts carefully assess all the documents, and all the healthcare providers involved are invited to present at the commission hearings. At the end of each session, the judges declare their decision. The patient or healthcare provider then have one month to protest the decision of the court. If no protest is filed, the decision is considered final. However, if either the patient or healthcare provider protests the decision, another commission with 5 professionals will be convened to re-evaluate the claim. This process can continue with commissions of 7, 9, and finally 11 professionals if the parties continue to dispute the ruling.

*The inclusion and exclusion criteria are not mentioned in the write up. Clarity is needed to determine the proportion of deliveries that turned out to be claims in the study period.*

Thank you for your comment. I added the inclusion and exclusion criteria.

**Results**: Authors didn’t revise the manuscript as expected. There are typos that need to be revised in the submitted version. There are overlapping tables on subsequent pages like 2. The titles could be improved to have ‘claims’ rather than groups! Significant findings with p-values < 0.05 can be made bold.

Thank you for your comment. I revised the result.

**Comments raised in previous review**

*I think it would nice to write more about the settings of the health facilities in the methods rather than introducing them in the result section for the first time.*

Thank you for your comment. I added to the method section regarding different types of hospital in Iran.

*It’s still not clear if it’s a document review or participant interviews.*

Thank you for your comment. The data collection was conducted by reviewing medicolegal documents.

*Please add a table to show the baseline characteristics of the participants. It’s not so clear who the other medical staffs that were involved in the study. What participant characteristic were more likely to make a claim? Prime gravida or multiparous?*

Thank you for your comment. I added a table regarding other medical staff. And also, regarding gravidity.

*Best in the introduction of the results section to mention how many deliveries occurred generally, how many SVDs, instrumental deliveries (vacuum or forceps) occurred in the study periods? Who assisted in the deliveries and the key aspects that contributed to a claim and a non-claim? How many closed or paid claims were reported?*

Thank you for your comment. You raise an important point. Unfortunately, we do not currently have definitive information regarding the total number of deliveries. More research would be needed to determine the precise total. We suggest that further study is required to accurately quantify the total number of deliveries.

*Were there any c/sections that were done with no indication?*

Thank you for your comment. We categorized cesarean sections with no medical indication as elective c-sections, which were conducted based on the mothers' request.

*I don’t see any IUFD in the table? Did you regard perinatal mortality as IUFDs? Otherwise, how did you capture early neonatal deaths?*

Thank you for your comment. Yes, I have added the number of intrauterine fetal deaths (IUFD) to the perinatal mortality.

*The two groups being compared that were mentioned to be similar are quite confusing. Did you mean among those that malpractice vs. cases with no malpractice or vaginal vs. c/section?*

Thank you for your feedback. I have updated the text to use the term "claims" instead of "groups" to improve clarity.

*What characteristics were reported among those whose claims were paid? Are these characteristics similar among those who had vaginal vs. caesarean delivery? Adding a*

Thank you for your comment. I added multivariable regression analysis to result.

I compared the data that we had extracted from the medicolegal documents and presented them in the table.

**Discussion**: Authors haven’t revised the manuscript as expected.

*Comments from previous review*:

It would be ideal to recap the overall aim of the study and mention the key significant findings before comparison with the other studies. Knowing the contribution of the other specialties in regards to the claims contributed by obs/gyn would have been so handy at this point.

Thank you for your comment. I added this point.

In the write up there are no gyn cases! So how would you conclude that obs has more legal claims than gyn even in the Iranian setting?

Thank you for your comment. This is not the conclusion of the study. We have mentioned this point to highlight the importance of claims related to delivery events.

Which region are you referring to with the CS rate of 48%? Please don’t assume that the readers see through your eyes.

Thank you for your comment. I revised it.

Mentioning “*Additionally, the rate of paid claims among emergency caesarean sections was similar to that of vaginal deliveries*” is not the best comparison. I think I would compare it to the elective cases as the participant characteristics would be different”.

Thank you for your comment. I revised it.

I find most of the comparisons in Europe! It would have been interesting to hear how neighbouring countries to Iran or Sub-Saharan Africa would compare in regards to medico-legal claims and obstetric cases.

Thank you for your comment. I added studies that are related to Iran and neihbering.

The strength of the study is not water-tight. Having it conducted in Iran doesn’t make it a strength in my view. Is it that there are many claims following c/s in Iran or because WHO thinks the C/s rate is higher than in other settings?

Thank you for your comment. I revised the strength according to your recommendation.

The limitations are not so clear as well. How would conducting a case-control study improve the study findings for your study? What did you mean by conduction the whole province evaluation would be a limitation? What challenges did you meet in executing this study?

Thank you for your comment. I revised the limitation based on your comment.

**Conclusion:** Authors haven’t revised the manuscript as per the expectation.

**Comments from previous review**

*In my evaluation, the claim that “this study revealed that claims related to deliveries were often associated with perinatal death” can not be drawn from the study findings.*

Thank you for your comment. I removed this sentence from conclusion.

*Though instrumental delivery is claimed to be a cause of malpractice, the authors make no mention of how many instrumental deliveries were reported among the claims. Clarity needs to be made in this regard.*

Thank you for your comment. I added this point to conclusion.

*Though the authors claim to have conducted the study to educate the specialists on medico-legal claims, there is no mention that there was a gap in the knowledge among the specialists. I request for recommendations that can be drawn from the study findings that can inform policy and future research.*

Thank you for your comment. I removed this sentence from conclusion.
