# Supplementary material for: Analysis of medico-legal claims related to deliveries: Caesarean section vs. vaginal delivery
Source: PLoS One. 2024 Nov 19;19(11):e0312614. doi: 10.1371/journal.pone.0312614 (PMC11575767; doi:10.1371/journal.pone.0312614)
Supplement: Attachment — Submitted filename: Reviewers comments to the Plos one manuscript_version 3.docx [file pone.0312614.s006.docx]

**Reviewer’s comments**: Dr Herbert Kayiga

**General comments:**

I take this privilege to appreciate authors for revising the manuscript. Most of the comments have been addressed in the current submission. Minor changes are required to ensure consistence in the write up. The grammar needs to be improved and very long sentences shortened. Add more references to the discussion. It’s more of what you found and less of how your findings compare to existing literature.

**Specific comments**:

**Abstract:**

**Introduction**: We can say that “The Iranian National Health Service (NHS) suggested…. Be consistent with the writing “caesarean section”. In some places you write ‘Cesarean

sections,”

**Introduction/Background**:

Kindly indicate year the global caesarean rate was taken.

**Discussion**: The write up has greatly improved but more references need to be added to beef up arguments. For example

“In a previous study conducted by Azimi et al. in Iran, they conducted an observational study on 1,116 medical claims in the field of obstetrics and gynecology over a two-year period”.

“Another study conducted in Italy revealed that approximately 30% of complaints related to childbirth resulted in payment

compensation. In consistent with our results, the majority of these claims were associated with caesarean section complications, extending beyond the surgical procedure itself”.

No references added.
