# Supplementary material for: Analysis of medico-legal claims related to deliveries: Caesarean section vs. vaginal delivery
Source: PLoS One. 2024 Nov 19;19(11):e0312614. doi: 10.1371/journal.pone.0312614 (PMC11575767; doi:10.1371/journal.pone.0312614)
Supplement: Attachment — Submitted filename: minor revision.docx [file pone.0312614.s007.docx]

Reviewer #1: Reviewer’s comments: Dr Herbert Kayiga
General comments:
I take this privilege to appreciate authors for revising the manuscript. Most of the comments have been addressed in the current submission. Minor changes are required to ensure consistence in the write up. The grammar needs to be improved and very long sentences shortened. Add more references to the discussion. It’s more of what you found and less of how your findings compare to existing literature.

Thank you so much for your Comments. I sincerely appreciate your valuable comments, which I believe have greatly helped to improve our study. I want to express my deep gratitude for your time and consideration.

I have revised the text and included additional relevant references.

Specific comments:
Abstract:
Introduction: We can say that “The Iranian National Health Service (NHS) suggested….

Thank you for your comment. I revised it.

Be consistent with the writing “caesarean section”. In some places you write ‘Cesarean
sections,”

Thank you for your comment. I revised it.

Introduction/Background:
Kindly indicate year the global caesarean rate was taken.

Thank you for your comment. I added the year.

Discussion: The write up has greatly improved but more references need to be added to beef up arguments. For example

“In a previous study conducted by Azimi et al. in Iran, they conducted an observational study on 1,116 medical claims in the field of obstetrics and gynecology over a two-year period”.

“Another study conducted in Italy revealed that approximately 30% of complaints related to childbirth resulted in payment
compensation. In consistent with our results, the majority of these claims were associated with caesarean section complications, extending beyond the surgical procedure itself”.

No references added.

Thank you for your comment. I added the references.

Reviewer #3: Researchers have conducted a suitable study for legal issues and complaints about cesarean section and vaginal delivery.

Such a conclusion cannot be drawn from the results of this study, and the findings do not match the conclusions.
Thank you for your comment. I revised the conclusion.

The limitations of the study are not mentioned in detail.

Thank you for your comment. I mentioned more detail for limitation.

Researchers should note that significant statistical difference and significant statistical significance always mean clinically significant difference and may not be clinically significant. It seems that this happened in this study.

Thank you for your comment. Our findings indicate that instrumental delivery is associated with an increased risk of paid claims, a trend also observed in previous studies. While this may evolve in future research, it could be clinically relevant.
